# Cell cycle-regulated transcription factor AP2XII-9 is a key activator for asexual division and apicoplast inheritance in *Toxoplasma gondii* tachyzoite

**DOI:** 10.1128/mbio.01336-24

**Published:** 2024-08-29

**Authors:** Yuehong Shi, Xuan Li, Yingying Xue, Dandan Hu, Xingju Song

**Affiliations:** 1 Guangxi Key Laboratory of Animal Breeding, Disease Control and Prevention, College of Animal Science and Technology, Guangxi University, Nanning, China; 2 Guangxi Zhuang Autonomous Region Engineering Research Center of Veterinary Biologics, Nanning, China; Albert Einstein College of Medicine, Bronx, New York, USA

**Keywords:** *Toxoplasma gondii*, ApiAP2, cell cycle, cell division, apicoplast, inner membrane complex, RON

## Abstract

*Toxoplasma gondii* is an intracellular parasitic protozoan that poses a significant risk to the fetus carried by a pregnant woman or to immunocompromised individuals. *T. gondii* tachyzoites duplicate rapidly in host cells during acute infection through endodyogeny. This highly regulated division process is accompanied by complex gene regulation networks. TgAP2XII-9 is a cell cycle-regulated transcription factor, but its specific role in the parasite cell cycle is not fully understood. In this study, we demonstrate that TgAP2XII-9 is identified as a nuclear transcription factor and is dominantly expressed during the S/M phase of the tachyzoite cell cycle. Cleavage Under Targets and Tagmentation (CUT&Tag) results indicate that TgAP2XII-9 targets key genes for the moving junction machinery (RON2, 4, and 8) and daughter cell inner membrane complex (IMC). TgAP2XII-9 deficiency resulted in a significant downregulation of rhoptry proteins and rhoptry neck proteins, leading to a severe defect in the invasion and egress efficiency of tachyzoites. Additionally, the loss of TgAP2XII-9 correlated with a substantial downregulation of multiple IMC and apicoplast proteins, leading to disorders of daughter bud formation and apicoplast inheritance and further contributing to the inability of cell division and intracellular proliferation. Our study reveals that TgAP2XII-9 acts as a critical S/M-phase regulator that orchestrates the endodyogeny and apicoplast division in *T. gondii* tachyzoites. This study contributes to a broader understanding of the complexity of the parasite’s cell cycle and its key regulators.

**IMPORTANCE:**

The intracellular apicoplast parasite *Toxoplasma gondii* poses a great threat to the public health. The acute infection of *T. gondii* tachyzoites relies on efficient invasion by forming a moving junction structure and also fast replication by highly regulated endodyogeny. This study shows that an ApiAP2 transcription factor, TgAP2XII-9, acts as an activator for the S/M-phase gene expression, including genes related to daughter buds and moving junction formation. Loss of TgAP2XII-9 results in significant growth defects and disorders in endodyogeny and apicoplast inheritance of the parasites. Our results provide valuable insights into the transcriptional regulation of the parasite cell cycle and invading machinery in *T. gondii*.

## INTRODUCTION

*Toxoplasma gondii*, an intracellular parasitic protozoan of the phylum Apicomplexa, can infect humans and almost all warm-blooded animals worldwide (1). It poses a significant risk to the fetus carried by pregnant women and immunocompromised individuals, causing severe complications and posing a public health concern worldwide (1). *T. gondii* has a complex facultatively heteroxenous life cycle comprising asexual and sexual stages. The asexual stage in the warm-blooded intermediate host consists mainly of fast-replicating tachyzoites and quiescent bradyzoites. Continuous asexual replication through endodyogeny of tachyzoites is the main cause of clinical symptoms in humans and animals (2); during endodyogeny, each tachyzoite in the host cell produces two daughter parasites via budding (3). The sexual reproductive cycle is restricted to the intestines of the felid definitive host, during which parasites are amplified by endopolygeny (4).

The growth of *T. gondii* tachyzoites in cells involves a complicated lytic cycle, including invasion, intracellular replication, and egress (2). Notably, the cell cycle of *T. gondii* can be categorized into G1, S, M, and C phases, lacking the classical definition of the G2 phase (5–7). During this process, the division and separation of tachyzoite organelles are allocated to the daughter cells in a highly coordinated manner, ensuring that each daughter parasite obtains a full set of organelles (8). Remarkably, the cell cycles of tachyzoites within the same parasitophorous vacuole are typically synchronized, revealing a precise and robust regulatory system to coordinate the synchronized division of these parasites (8). Transcriptomic analysis has shown differences in the gene expression at different developmental stages or cell cycles (6). In *T. gondii*, there is a “just-in-time” expression pattern in which the parasite produces transcripts and proteins of certain genes through strict regulation when they are required for their function (7, 9).

Apicomplexan parasites have a unique family of transcription factors that are characterized by the possession of one or more plant-like AP2 DNA-binding domains. These apicomplexan AP2 transcription factors (ApiAP2) can bind to specific promoter sequences and regulate the expression of target genes (10). A total of 67 ApiAP2s have been identified in *T. gondii*, of which 24 are considered to be cell cycle-regulated ApiAP2s (9). TgAP2XI-4, TgAP2IX-4, TgAP2IV-3, TgAP2IV-4, and TgAP2IX-9 have been shown to be involved in the regulation of gene expression and the formation of tissue cysts (11–15). TgAP2X-4 is crucial for the growth of *T. gondii* during the acute stage of infection and is able to regulate the expression of cell cycle genes in tachyzoites (16). TgAP2X-5 has been proven to indirectly regulate the promoter of virulence genes expressed in the S/M phase, mainly in synergy with TgAP2XI-5 (7). TgAP2IX-5 was found to be a key transcriptional regulator of the asexual cell cycle and plastid division in *T. gondii* (17, 18). Knockdown of the cell cycle-regulated TgAP2IX-5 also results in flexibility of the cell cycle pattern from endodyogeny to endopolygeny (17). Chromatin immunoprecipitation results demonstrated that TgAP2IX-5 binds to the promoters of many of the inner membrane complex (IMC) genes and five cell cycle-regulated ApiAP2s (TgAP2IV-4, TgAP2III-2, TgAP2XII-9, TgAP2X-9, and TgAP2XII-2) (19). The individual gene knockdown experiment showed that TgAP2III-2 is dispensable, while TgAP2IV-4, TgAP2X-9, TgAP2XII-2, and TgAP2XII-9 may be essential genes (14). TgAP2IV-4 was further identified as a key suppressor of bradyzoite genes, and deletion of this gene results in the expression of a subset of bradyzoite-specific proteins during the replication of tachyzoites (14). TgAP2XII-2 is associated with TgAP2IX-4 and microrchidia (MORC) and represses merozoite-specific gene expression (20, 21). TgAP2X-9 is repressed by TgAP2IX-5 (19) and may be phosphorylated by the CDK-related kinase TgCrk4 in the control of the G_2_ phase (22). However, the role of TgAP2XII-9 remains unclear.

In this study, we identify TgAP2XII-9 as an important S/M-phase transcription factor that activates the transcription of key genes of the moving junction complex and IMCs during daughter cell formation. Knockdown of TgAP2XII-9 leads to significant growth defects as well as disorders in endodyogeny and apicoplast inheritance. Our results provide valuable insights into the transcriptional regulation of the parasite cell cycle and invading machinery in *T. gondii*.

## RESULTS

### TgAP2XII-9 expression in the nucleus during the S/M phase of the cell cycle in tachyzoites

**Fig 1:**
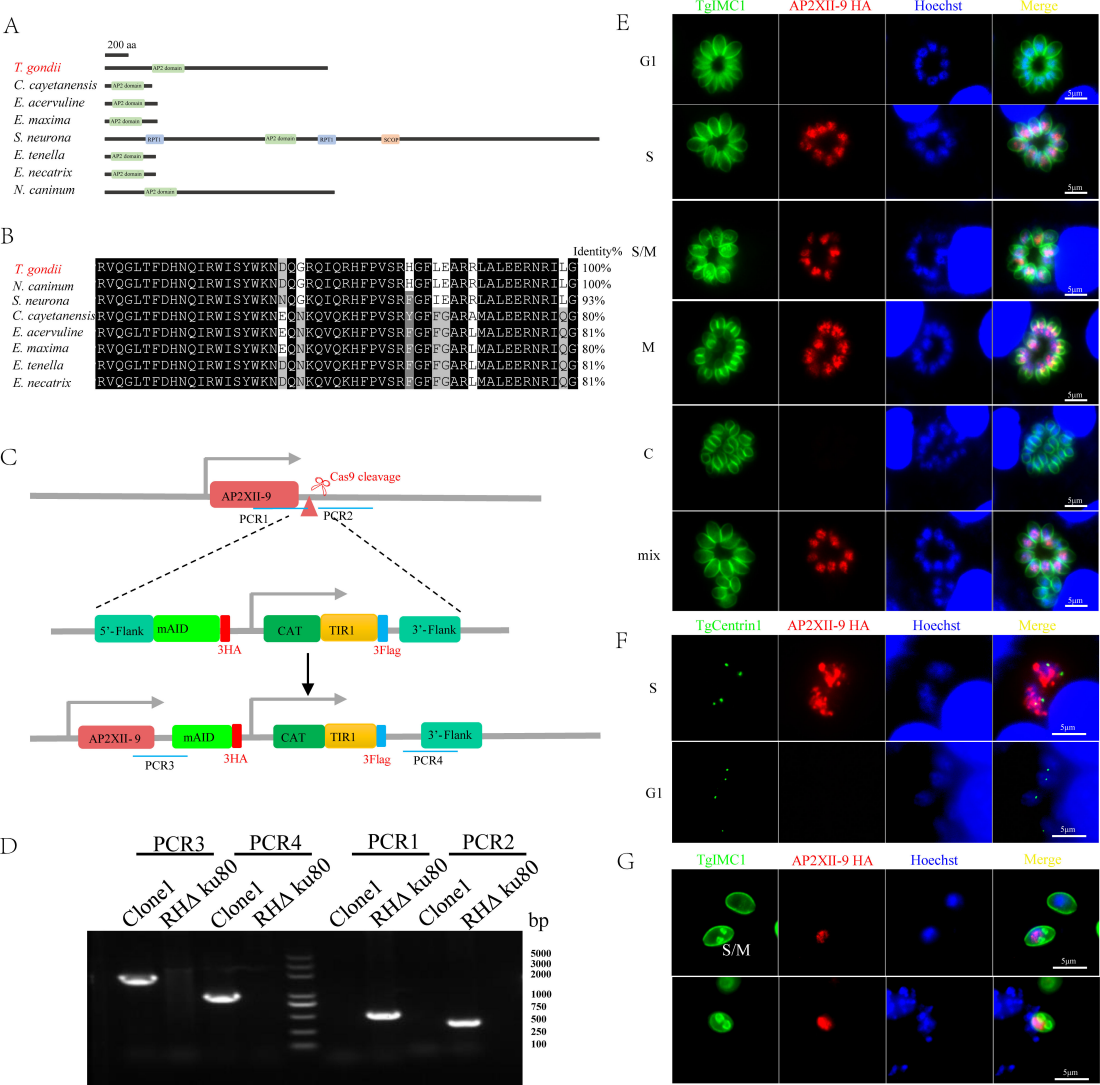
TgAP2XII-9 is a cell cycle-regulated protein. (**A**) Schematic representation of the conserved AP2 domain-containing proteins from *T. gondii* (TGGT1_251740), *Cyclospora cayetanensis* (cyc_03452), *Eimeria acervulina* (EAH_00021030) and *Eimeria maxima* (EMWEY_00029950), *Sarcocystis neurona* (SN3_01800170), *Eimeria tenella* (ETH2_0411800) and *Eimeria necatrix* (ENH_00052610), and *Neospora caninum* (NCLIV_066800). Domains are predicted by SMART (25). (**B**) Multiple sequence alignments of the AP2 domains in apicomplexan parasites. The AP2 domain sequences from TgAP2XII-9 were aligned with their homologs. Regions of high identity and similarity between AP2 domain sequences are shown as black and gray columns, respectively. The percent homology of TgAP2XII-9 with each AP2 domain is shown at the end of the alignment. (**C**) Diagram showing the strategy for tagging TgAP2XII-9 with AID-3HA in wild-type parasites. The location of the primers used for the integration PCR is indicated. (**D**) Confirmation of recombinant and clonal lines of iKD TgAP2XII-9 parasites by PCR. (**E and F**) Subcellular localization of the TgAP2XII-9 protein during the tachyzoite cell cycle. The cell cycle stage is shown on the left side of the images. Tachyzoites were co-stained using mouse anti-HA (red), rabbit anti-TgIMC1 (green), or rabbit anti-TgCentrin1 (green) antibodies. Hoechst 33258 was used to stain nuclei. IMC1 staining was used to demonstrate the tachyzoite cell cycle. Division of the centrosome initiated during the S phase was identified by staining of TgCentrin1. Parasites in the G1 phase contain single centrosomes, whereas those in the S-phase are duplicated. C, cytokinesis; G1, gap phase; M, mitotic phase; S, synthesis phase. (**G**) Representative images of TgAP2XII-9 expression in extracellular parasites. Hoechst 33258 was used to stain nuclei. TgIMC1 staining was used to demonstrate the tachyzoite cell cycle. Scale bars = 5 µm.

Among a total of 67 T. *gondii* ApiAP2s, TgAP2XII-9 (TGGT1_251740, https://toxodb.org) was recognized as a potential cell cycle-related ApiAP2 transcription factor (9, 23, 24). Amino acid sequence analysis showed that TgAP2XII-9 contains one conserved AP2 domain located at the 402–455 amino acids of its N-terminus. The AP2 domain of TgAP2XII-9 shares over 80% amino acid homology with other apicomplexans, including *Cyclospora cayetanensis* (cyc_03452), *Eimeria acervulina* (EAH_00021030), *Eimeria maxima* (EMWEY_00029950), *Sarcocystis neurona* (SN3_01800170), *Eimeria tenella* (ETH2_0411800) and *Eimeria necatrix* (ENH_00052610), and *Neospora caninum* (NCLIV_066800) (Fig. 1A and B). A previous transcriptomics study demonstrated that the expression of TgAP2XII-9 is characterized as highly dynamic during the parasite cell cycle, with peak expression during the S/M phase (9) (https://toxodb.org). To confirm that the TgAP2XII-9 protein is expressed during the cell cycle, we strategically fused the mAID-3HA tag to the C-terminal end of its endogenous locus, enabling tagging under their native promoters (Fig. 1C). The correct insertion of endogenous tags and single clones was confirmed by polymerase chain reactions (PCRs) (Fig. 1D). Immunofluorescence assays (IFA) using cell cycle markers demonstrated that TgAP2XII-9 is located in the nucleus and is mostly expressed in the S/M phase (Fig. 1E and F). The expression of TgAP2XII-9 was detected during centrosome divisions rather than in individual centrosomes (identified by TgCentrin1; a marker of the outer core of the centrosome, Fig. 1F), indicating that TgAP2XII-9 is expressed in the S phase rather than the G1 phase. Moreover, the expression of TgAP2XII-9 was also detected in the M phase, as indicated by the budding marker TgIMC1 (Fig. 1E). In addition, extracellular tachyzoites were also stained to investigate the expression of the TgAP2XII-9 protein, and the results showed that TgAP2XII-9 was expressed mainly in the nucleus during daughter bud formation (Fig. 1G). Therefore, the expression of the TgAP2XII-9 protein is rigorously controlled by the cell cycle.

### TgAP2XII-9’s critical role in tachyzoite growth and intracellular replication

**Fig 2:**
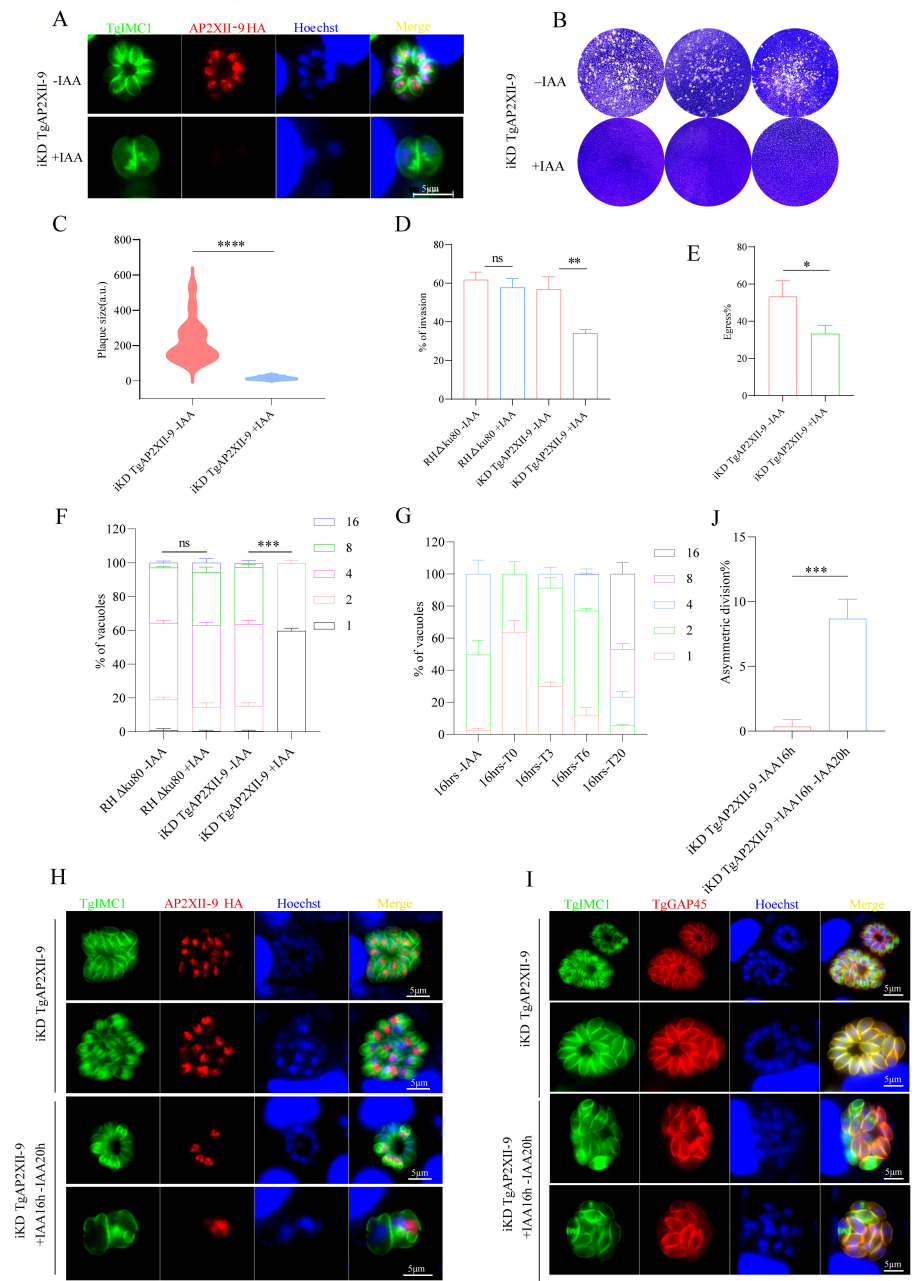
TgAP2XII-9 is essential for the replication of *T. gondii* tachyzoites. (**A**) Fluorescence microscopy of intracellular iKD TgAP2XII-9 parasites after treatment with 500 µM IAA or vehicle. Tachyzoites were co-stained with mouse anti-HA (red) and rabbit anti-TgIMC1 (green) antibodies. Nuclei were stained with Hoechst (blue). Scale bars = 5 µm. (**B and C**) Plaque assay of iKD TgAP2XII-9 parasites grown on IAA- or vehicle-treated HFF cells for 7 days. Plaque areas were measured and counted by Photoshop C6S software (Adobe, USA). (**D**) Invasion efficiency of indicated parasites that were pretreated with or without IAA for 48 hours. Then, infect HFF cells for 1 hour at 37°C. Data represent the mean ± SD of three independent experiments, each counting 15 fields per strain, analyzed by unpaired two-tailed Student’s *t*-test. (**E**) Efficiencies of A23187-induced egress of iKD TgAP2XII-9 parasites that were pretreated with or without IAA for 30 hours. The average number of ruptured PVs was determined by randomly counting 100 vacuoles per slide. Means ± SD of three independent experiments were graphed. (**F**) Intracellular parasite replication of the indicated strains’ incubation with IAA or vector for 24 hours (added 1 hour post-invasion). Data are expressed as the mean ± SEM of three independent assays, each counting 100 total PVs per strain. (**G**) Assessment of intracellular growth of iKD TgAP2XII-9 parasites after 16 hours of culture in the IAA-added medium (added 1 hour post-invasion), followed by 0, 3, 6, and 20 hours of culture in the standard medium (16 hours - T0/T3/T6/T20). Parasite nuclei were stained with Hoechst 33258, and the parasite IMC was stained with the anti-TgIMC1 antibody. The number of tachyzoites in the PV was counted by three independent assays of 100 PVs each. (**H and I**) Immunofluorescence assay of iKD TgAP2XII-9 parasites treated with IAA for 16 hours (added 1 hour post-invasion) and recovery of growth after IAA washout. Parasites were fixed and stained with rabbit anti-TgIMC1 antibody (green), mouse anti-HA antibody (red), or rabbit anti-TgGAP45 antibody (red). (**J**) The abnormal proportion of asynchronously dividing parasitic vacuoles. *: *P* < 0.05, **: *P* < 0.01, ***: *P* < 0.001, and ****: *P* < 0.0001; n.s: no significant difference.

To assess the functions of TgAP2XII-9 in *T. gondii*, an indole-3-acetic acid (IAA) degradation (mAID) system (26, 27) was used to generate an inducible knockdown (iKD) of TgAP2XII-9 parasites (iKD TgAP2XII-9) (Fig. 1C and D). Immunofluorescence assays confirmed that the addition of IAA resulted in the degradation of the TgAP2XII-9 protein in tachyzoites (Fig. 2A). To comprehensively evaluate the viability of TgAP2XII-9-deficient parasites, we monitored the formation of plaques during a continuously maintained 7-day culture. The results showed that the parasites were unable to form plaques in host cells upon IAA treatment, whereas untreated parasites formed noticeable plaques (Fig. 2B and C). The growth of *T. gondii* tachyzoites in cells involves a complicated lytic cycle, including invasion, intracellular replication, and egress. The reduction in plaque formation may be caused by impairment of one or more steps of the lytic cycle. Thus, we next sought to investigate the role of TgAP2XII-9 in the lytic cycle biology of *T. gondii*. For the invasion assay, iKD TgAP2XII-9 parasites pretreated with or without IAA for 48 hours were allowed to invade HFF cells for 1 hour, and then the invasion efficiency was determined by a two-color staining assay. The iKD TgAP2XII-9 parasites showed a significant reduction (34%, *P* < 0.01) compared to untreated parasites (Fig. 2D). Subsequently, the calcium ionophore A23187 was used to assess the egress ability of the parasites, and the results showed a significant decrease in the egress ratio (33.3%, *P* < 0.05) in the iKD TgAP2XII-9 parasites after 30 hours of IAA treatment (Fig. 2E). For intracellular replication, degradation of TgAP2XII-9 led to a severe decrease in the intracellular replication capacity of parasites having one or two tachyzoites per vacuole, whereas parasites without IAA treatment mostly had four and eight tachyzoites per vacuole (Fig. 2F). It is worth noting that the replication defects of the parasites caused by degradation of the TgAP2XII-9 protein are not irreversible. After the addition of IAA treatment for 16 hours to induce degradation of the TgAP2XII-9 protein, the parasites were placed in a culture medium without IAA for another 0, 3, 6, and 20 hours. The results showed that the proliferation ability of parasites treated in this way significantly recovered during this period (Fig. 2G). Interestingly, about 8.7% of the parasitic vacuoles (PVs) exhibited significant asynchronous division after removal of IAA (*P* < 0.001, Fig. 2H through J), implying inconsistent growth cycles of parasites within the same PV. These results indicate that TgAP2XII-9 expression is crucial for the lytic cycle of tachyzoites.

### TgAP2XII-9 regulates the transcription of periodic genes and targets ROP and IMC genes

**Fig 3:**
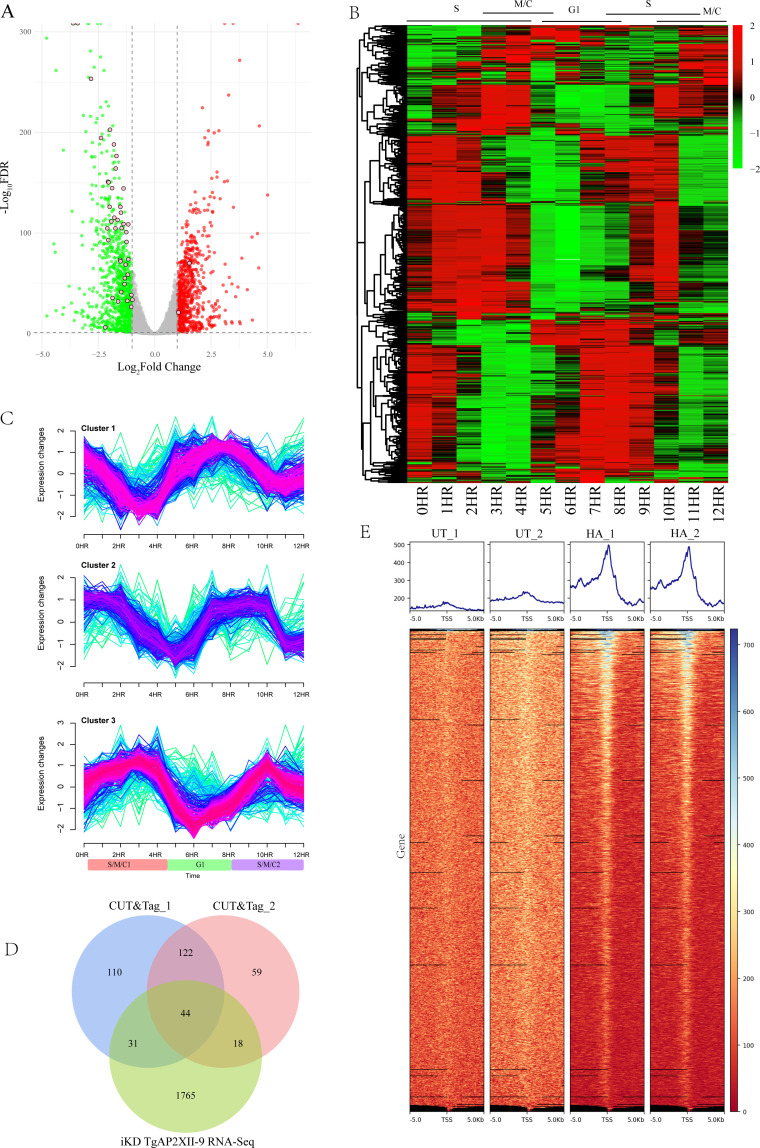
TgAP2XII-9 controls multiple cell cycle-regulated genes. (**A**) Volcano plots showing differentially expressed genes after degradation of the TgAP2XII-9 protein by IAA treatment for 24 hours (*n* =  8,920). Green and red dots indicate significantly downregulated and upregulated genes, respectively. The pink and circled dots represent CUT&Tag hits. (**B**) Heatmap showing the cell cycle expression of all individual transcripts downregulated in the iKD TgAP2XII-9 strain during 24 hours of IAA treatment. Cell cycle phases (**G1-S-M-C**) are shown at the top. (**C**) Mfuzz cluster analysis illustrating changes in the expression of downregulated genes during cell cycle progression (9). Fuzzy c-means clustering identified three distinct temporal patterns of the protein expression. The x-axis represents time points in hours, while the y-axis represents the log_2_-transformed, normalized intensity ratios in each stage. Cluster 1: G1-S high expression peaks; Cluster 2: S-M low expression peaks; Cluster 3: G1 low expression peaks. (**D**) Venn diagram of TgAP2XII-9-dependent DEGs intersected with the TgAP2XII-9 CUT&Tag genes. (**E**) Profile and heat maps of the averaged sum showing the CUT&Tag called the peaks of TgAPXII-9 (HA) around the TSS of the parasite genes. The top panels show the average signal profile of the genomic loci centered on the TSS (± 5 kb). The lower panels show heat maps of peak density around the same genomic loci. The color scale used to interpret the signal intensity is located on the right side of each graph.

Potentially regulated genes of the transcription factor TgAP2XII-9 were investigated by RNA-seq using TgAP2XII-9 iKD parasites. A total of 1,858 differentially expressed genes (DEGs) were identified after TgAP2XII-9 knockdown, including 1,017 downregulated genes (more than twofold decrease in the transcript level; *P* < 0.05) and 841 upregulated genes (more than twofold decrease in the transcript level; *P* < 0.05) (Fig. 3A; Table S1). The cell cycle expression profile of downregulated genes was analyzed using previously published data (9) and represented as a heat map (Fig. 3B). The results showed that most of the downregulated genes exhibited features of cell cycle regulation. Subsequently, we clustered these downregulated genes with similar expression trends and found that the expression profiles of these genes could be divided into three clusters (Fig. 3B and C; Table S1). Interestingly, the genes in cluster 3 showed the highest expression in S and M phases and the lowest expression in the G1 phase, which is consistent with the expression patterns of TgAP2XII-9. On the contrary, most of the genes in cluster 1 showed highest expression in the G1 phase and lowest expression in the S and M phases. Most genes in cluster 2 showed peak expression in late G1 and early S phases, whereas the low expression peaks corresponded to those of M and early G1 phases (Fig. 3C). We also noticed that there are several enteroepithelial stage (EES)-specific genes altered after TgAP2XII-9 knockdown, including 111 upregulated and 97 downregulated (Fig. S1).

To further characterize the direct target genes of TgAP2XII-9, CUT&Tag experiments were performed using the endogenously 3xHA tagged parasite and RH strain (Fig. 3D). Promoters of 251 and 320 genes were enriched for the two individual CUT&Tag experiments, respectively (Fig. 3E; Table S1). Of these, 164 genes appeared in both replicates, with a total of 44 genes that were differently expressed after TgAP2XII-9 knockdown. Of these 44 potential targets, eleven proteins were rhoptry proteins (ROP1, ROP4, ROP15, ROP17, ROP39, ROP47, and ROP48) or rhoptry neck proteins (RON2, RON3, RON4, and RON8); 14 IMCs or glideosome-associated proteins, namely, IMC3, IMC4, IMC10, IMC16, IMC31, IMC34, AC2, AC12, AC13, GAP40, GAP50, GAPM3, GAPM1A, GAPM2A, and IMC localizing protein (ILP1). Moreover, all these ROPs/RONs and IMC-related genes were downregulated upon TgAP2XII-9 depletion, indicating that TgAP2XII-9 binds directly to the promoters of these genes and functions as an activator.

We also investigated the potential interactome of TgAP2XII-9 by immunoprecipitation and MS-based quantitative proteomics using iKD TgAP2XII-9 strain and wild-type with the anti-HA antibody. A total of 36 proteins were significantly enriched (and 32 proteins uniquely co-precipitated) in the iKD TgAP2XII-9 strain (Table S2), including three ApiAP2s (TgAP2XII-4, TgAP2XII-1, and TgAP2X-7). These interactions should be validated further.

### TgAP2XII-9 influences the invasion and egress efficiency of tachyzoites by regulating the expression of ROP genes

**Fig 4:**
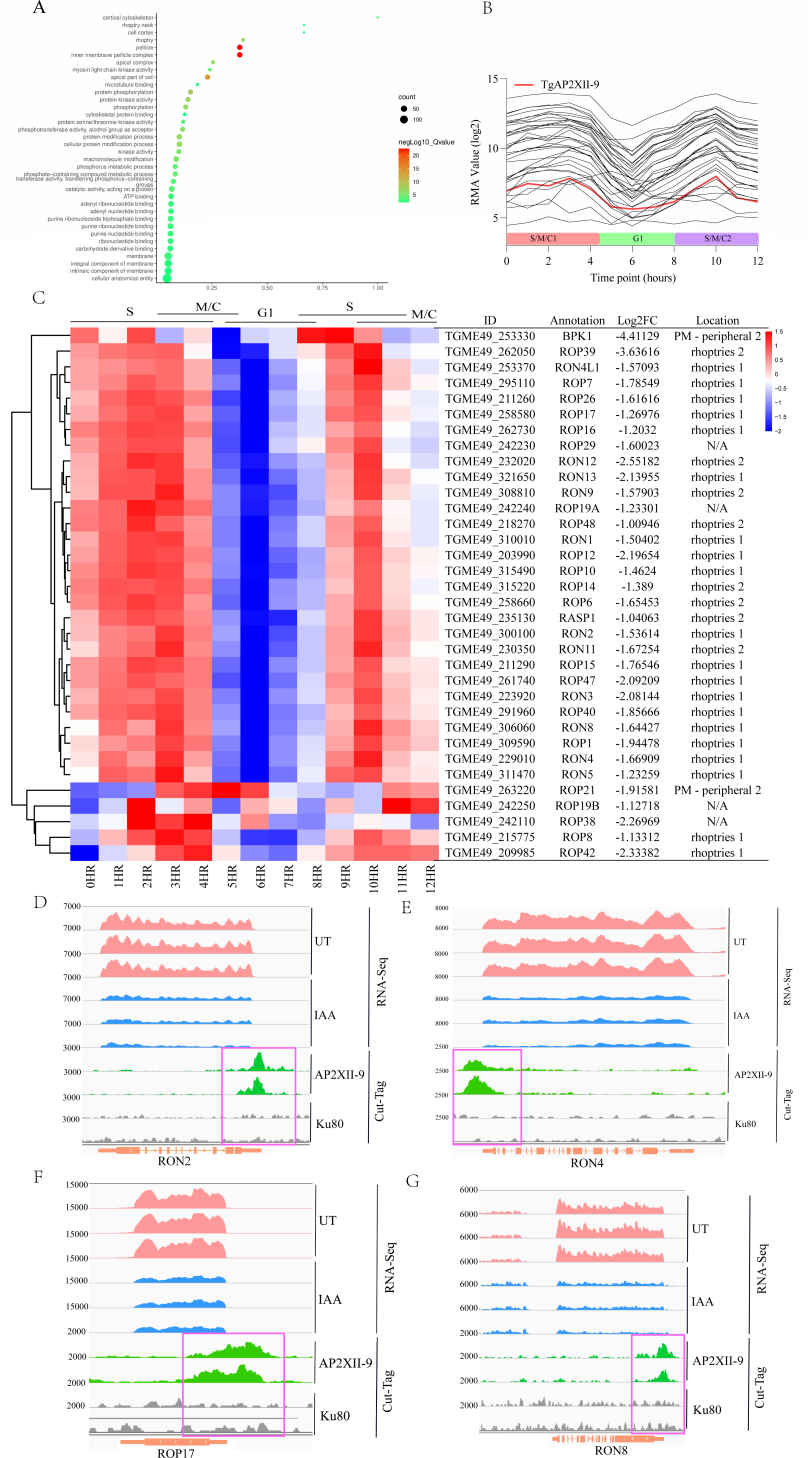
TgAP2XII-9 is required for efficient invasion of host cells. (**A**) Performance of functional enrichment for cluster 3 proteins by gene ontology (GO) analysis using ToxoDB annotations. Significant GO terms are shown (Benjamini < 0.1). (**B**) The mRNA profiles of the downregulated ROP and RON genes in TgAP2XII-9 depleted parasites according to the cell cycle expression profiles (9) (source of data: ToxoDB.org); RMA, robust multiarray average. (**C**) Hierarchical clustered transcriptional heatmap showing the transcription levels of downregulated ROP and RON genes (*n* = 35) after TgAP2XII-9 depletion at different cell cycle phases. The top of the heatmap shows the expression scale of cell cycle phases. IGV screenshots of the genomic regions of RON2 (**D**), RON4 (**E**), ROP17 (**F**), and RON8 (**G**). CUT&Tag profiles were obtained using antibodies directed against HA (TgAP2XII-9 tagged) in chromatin sampled from the TgAP2XII-9-mAID-HA strain and the RH Δku80 strain. RNA-seq data from iKD TgAP2XII-9 parasites after 24 hours of treatment with IAA or vehicle are shown in blue and pink peaks, respectively. The normalized RPKM for CUT&Tag and RNA-seq reads are shown on the y-axis.

As TgAP2XII-9 knockdown results in differential transcription of multiple periodic genes, these genes can be categorized into three major clusters. Our attention was first focused on the genes in cluster 3 because its expression pattern is similar to that of TgAP2XII-9, both of which are highly expressed in the S/M phase and downregulated in the G1 phase. GO enrichment analysis revealed that cluster 3 genes were mainly distributed in the inner member pellicle complex, pellicle, and apical part of the cell (Fig. 4A). We further conducted a detailed analysis of these genes and found that most of the proteins clustered in the apical part of the cell were ROP and RON proteins (*P*-value < 3.26E-15). These ROP and RON proteins have a very similar periodic expression pattern to that of TgAP2XII-9 during the cell cycle, peaking at S and M phases and reaching a minimum at the G1 phase (Fig. 4B and C).

The moving junction (MJ), which consists of apical membrane antigens and RON protein complex, is a key structure for parasite invasion into host cells (28). The deletion of TgAP2XII-9 resulted in a significant downregulation of 11 RONs. As a result, the transcription levels of all moving junction-related RONs (RON2, RON4, RON4L1, RON5, and RON8) were significantly downregulated. In addition, the promoters of ROP17, RON2, RON4, and RON8 were directly bound by TgAP2XII-9, as shown in the CUT&Tag results (Fig. 4D through G). The formation of MJ in the tachyzoite is characterized by the interaction of AMA1 (secreted by micronemes and relocalized to the parasite surface prior to invasion) and the export of RON2, with further recruitment of other RONs (29). The depletion of these RONs would greatly impair the invasion ability, and we did observe a significant reduction in the invasion rate after TgAP2XII-9 depletion. Interestingly, the bradyzoite MJ components AMA2 (Log_2_fold change = −1.41; FDR = 6.48E-22) and AMA4 (Log_2_fold change = −4.37; FDR = 2.25E-262), which are key proteins for cyst burden during the onset of chronic infection (30), were also downregulated in the absence of TgAP2XII-9. This suggests that the invasion ability of bradyzoites may also be affected by TgAP2XII-9, but further validation is needed.

We also identified 23 rhoptry proteins that are downregulated upon deletion of TgAP2XII-9 and seven ROPs whose promoters are directly bound by TgAP2XII-9 informed from the CUT&Tag peaks. These genes, including ROP1 (31), ROP4, ROP15, ROP17 (32, 33), ROP39 (34), ROP47, and ROP48 (35), are reported to be virulence factors or involved in host–parasite interactions. In summary, our results demonstrate that TgAP2XII-9 controls the expression of core genes for MJ and parasite virulence factors, which suggests that TgAP2XII-9 contributes to parasite invasion and virulence.

### TgAP2XII-9 controls the expression of multiple IMC genes and is crucial for the formation of daughter buds

In addition to the ROP protein, a large number of cluster 3 genes were also enriched in the inner member pellicle complex and pellicle (Fig. 4A), and the majority of these genes were found to be genes encoding IMC proteins of tachyzoites (*P*-value < 2.15E-23). The IMC is an important organelle in *T. gondii* that plays a key role in parasite motility, invasion, egress, and replication (2, 36). IMC can be classified as proteins that are specifically localized to the maternal IMC, the daughter bud IMC, or both (37).

**Fig 5:**
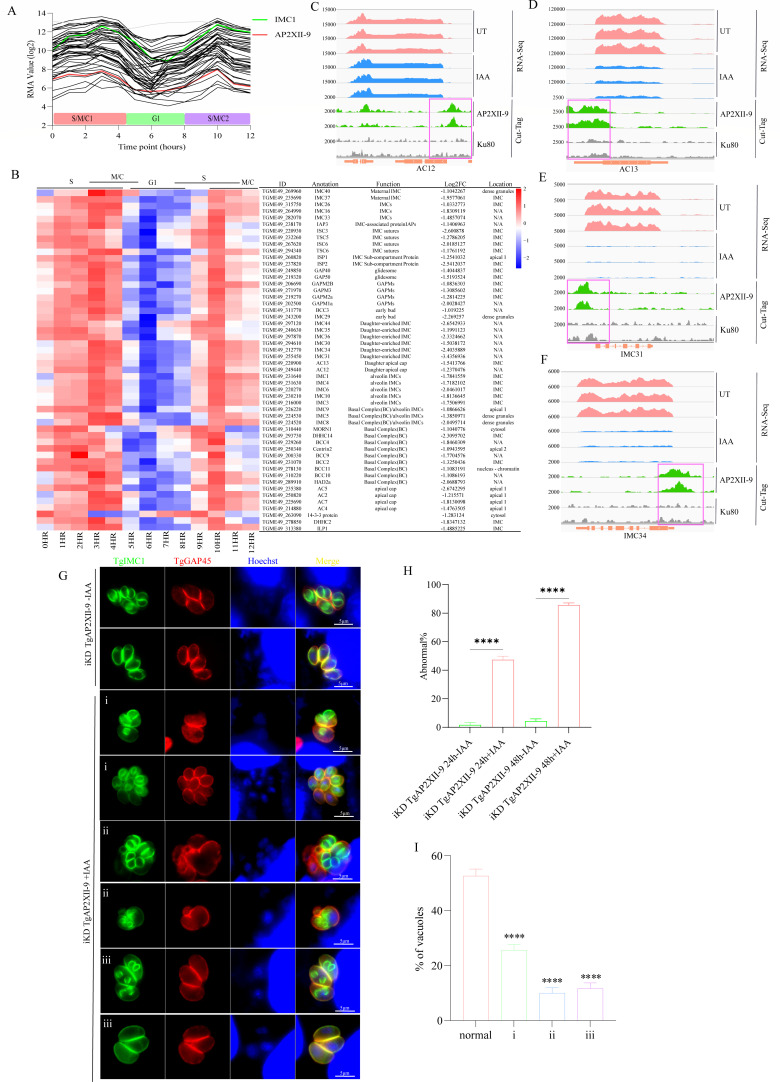
TgAP2XII-9 controls the expression of key genes involved in daughter parasite formation. (**A**) The mRNA expression showing downregulation of IMC genes after TgAP2XII-9 depletion (Log_2_FC < −1; *P*-value  <  0.01). Their transcript was abundant in various cell cycles of parasites; RMA, robust multiarray average. (**B**) Heatmap of the cell cycle expression profile for 52 IMC-associated genes transcripts downregulated (Log_2_FC  < −1; *P*-value  <  0.01) in TgAP2XII-9-deficient parasites. Scale of the cell cycle phase is shown at the top of the heatmap. IGV screenshots of the genomic region of AC12 (**C**), AC13 (**D**), IMC31 (**E**), and IMC34 (**F**). CUT&Tag profiles were obtained using antibodies directed against HA (TgAP2XII-9-tagged) in chromatin samples of the iKD TgAP2XII-9 strain and the RH Δku80 strain. RNA-seq data from iKD TgAP2XII-9 strain parasites after 24 hours of treatment with IAA or vehicle are shown in blue and pink peaks, respectively. The normalized RPKM for CUT&Tag and RNA-seq reads are shown on the y-axis. (**G**) IFA showing the patterns of daughter cell budding (stained with TgIMC1) in iKD TgAP2XII-9 parasites treated with or without IAA for 24 hours or 48 hours. Different patterns of abnormal budding were observed: (i) non-synchronous division, (ii) endopolygeny, and (iii) the appearance of an odd number of tachyzoites in a PV. Nuclei were stained with Hoechst (blue). Scale bars = 5 µm. (**H**) Statistical analysis of the percentage of abnormally divided PVs after 24 hours and 48 hours of treatment with IAA (added 1 hour post-invasion). (**I**) Statistical analysis of the percentage of PVs in each type of abnormal divisions (types i, ii, and iii) compared to the total abnormal PVs after 24 hours of treatment with IAA. Means ± SD of three independent experiments, *: *P* < 0.05, **: *P* < 0.01, and ***: *P* < 0.001.

Depletion of the TgAP2XII-9 protein resulted in the downregulation of 52 IMC proteins (more than a twofold decrease in the transcriptional level; *P* < 0.05), exhibiting a cell cycle-regulated expression pattern consistent with that of TgAP2XII-9, with peak expression in S and M phases and reaching the lowest expression in the G1 phase (Fig. 5A and B). Among these downregulated IMC proteins, more than 10 proteins are known to be localized in daughter tachyzoites, namely, IMC1, IMC3, IMC6, IMC29, IMC30, IMC31, IMC34, IMC35, IMC36, IMC44, AC12, AC13, ISP1, and ISP2 (Fig. 5B) (37–41). Of these, seven proteins (IMC29, IMC30, IMC31, IMC34, IMC35, IMC36, and IMC44) are restricted to the IMC body of daughter buds, and two proteins (AC12 and AC13) are localized only to the apical cap of daughter buds (37). IMC29 has been identified as an important early daughter bud component for replication (37). CUT&Tag results also showed that the promoters of daughter cell-restricted AC12, AC13, IMC31, and IMC34 bind directly to TgAP2XII-9 (Fig. 5C through F).

Considering that the depletion of TgAP2XII-9 resulted in a significant downregulation of a large number of IMC proteins related to the formation of daughter buds in *T. gondii,* we further used TgIMC1 as a marker to verify whether the division of the TgAP2XII-9 deficient parasites was affected. After 24 and 48 hours of treatment with IAA, the iKD TgAP2XII-9 parasites exhibited disordered daughter divisions (Fig. 5G and H). Three different types of unnatural divisions were mainly observed: (i) nonsynchronous division characterized by different budding states (budding, no budding, just budding, and complete daughter cell formation) of tachyzoites in the same PV; (ii) endopolygeny with more than two daughter buds within a parasite (>2 daughter buds per maternal parasite); and (iii) the presence of an odd number of tachyzoites in a PV (Fig. 5G). Among them, the proportion of abnormal divisions was 26%, 10%, and 12% in cases i, ii, and iii, respectively, after 24 hours of treatment with IAA (Fig. 5I). When the IAA treatment was extended to 48 hours, the parasites were observed to grow continuously, and these defects seemed to accumulate over time. Quantification showed 86% of abnormal vacuoles after 48 hours of IAA treatment (Fig. 5H). These results indicate that TgAP2XII-9 significantly impairs the division of tachyzoites by regulating the transcription of various budding-related IMC genes, which ultimately leads to limited intracellular replication and egress defects in parasites.

### TgAP2XII-9 regulates apicoplast gene expression and its division

According to the cell cycle-regulated expression profile, the genes downregulated in TgAP2XII-9 deletion parasites were categorized into three clusters (clusters 1–3) (Fig. 3C). Previous GO analysis of the cluster 3 gene showed enrichment in IMC, ROP, and RON proteins (Fig. 4A). The downregulation of these genes may be responsible for abnormal division, intracellular replication stagnation, and reduced invasion and egress efficiency in the parasites. Considering that the expression peaks of genes from cluster 1 to cluster 3 progressed over time, we speculate that TgAP2XII-9 may directly or indirectly regulate gene expression in clusters 1–3.

**Fig 6:**
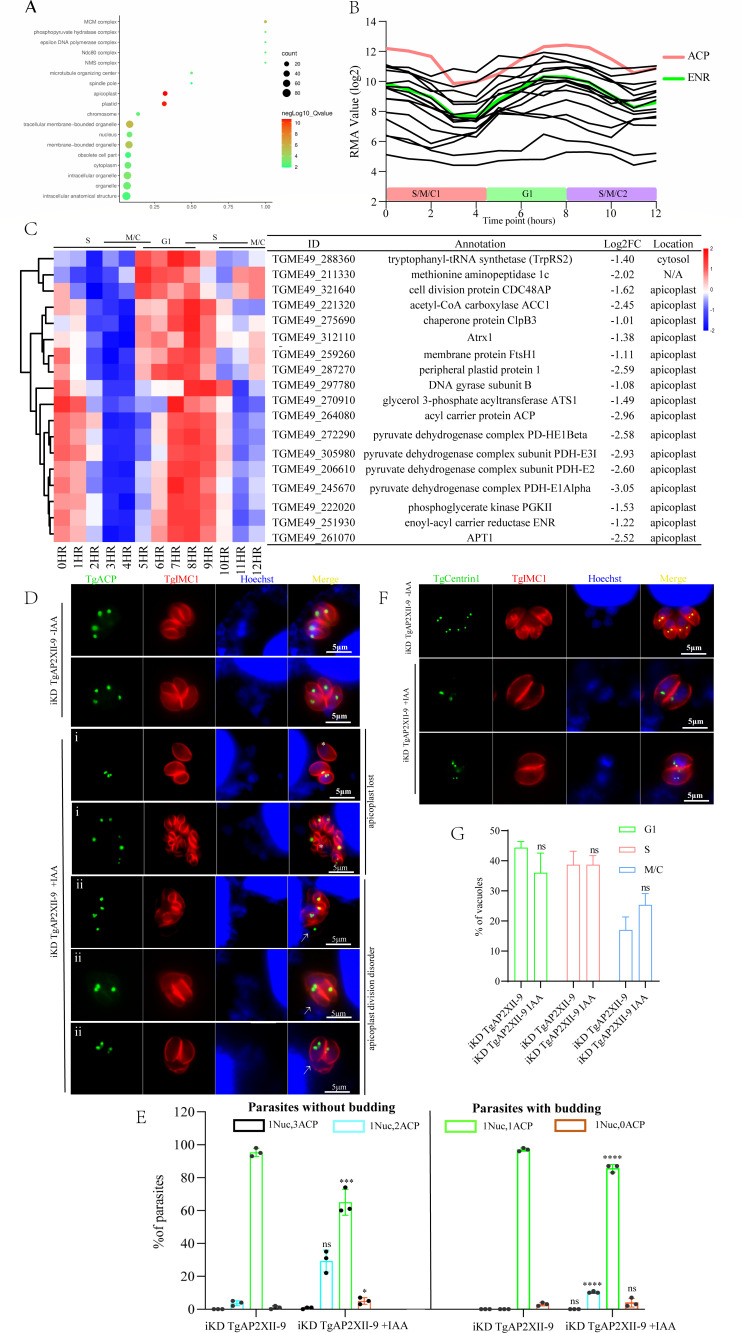
TgAP2XII-9 deficiency influences the expression of apicoplast genes. (**A**) GO analysis of cluster 1 protein in TgAP2XII-9-deficient parasites using ToxoDB annotations; significant GO terms are shown (Benjamini < 0.1). (**B**) Cell cycle expression patterns of 18 apicoplast genes downregulated in TgAP2XII-9-deficient tachyzoites (9). RMA, robust multiarray average. (**C**) Heatmap showing downregulation of 18 apicoplast genes after TgAP2XII-9 depletion. The putative annotation and location of these genes are listed. (**D**) Immunofluorescence analysis of apicoplast division in iKD TgAP2XII-9 parasites treated with or without IAA. Examples of different types of apicoplast abnormal divisions (shown by ACP staining) were observed. (i) Loss of apicoplast—asterisks mark parasites that produce 0 apicoplast; (ii) apicoplast division disorder—arrows indicate three or two apicoplasts in a daughter cell and one apicoplast in another daughter cell. TgIMC1 (red) serves as a daughter cell budding marker. The nucleus was stained with Hoechst (bar  =  5 µm). (**E**) Distribution of parasites containing the indicated number of segregated apicoplasts, as shown by Hoechst and TgACP staining on iKD TgAP2XII-9 parasites treated with or without IAA for 24 hours. 1 Nuc indicates one nucleus; 0, 1, 2, and 3 ACP indicates loss of apicoplast, one apicoplast, two apicoplasts, and three apicoplasts, respectively. Parasites with or without daughter cell budding (determined by IMC1 staining) were plotted separately. Hundred parasites with or without daughter cell budding were analyzed in each biological replicate. (**F**) Observation of centrosome division by IFA in iKD TgAP2XII-9 parasites treated with or without IAA. IFA was performed using antibodies to TgIMC1 (red) and TgCentrin1 (green). Hoechst was used to stain the nucleus. Parasites in the G1 phase contain single centrosomes, whereas parasites in the S-phase have duplicate centrosomes. The scale bar is shown on the lower right side of each image. (**G**) Quantification of phenotypes in 24 h-treated iKD TgAP2XII-9. At least 100 vacuoles were counted per biological replicate. Each experiment was repeated three times. Results were shown as means ± SD of three independent experiments, and unpaired two-tailed Student’s *t*-test were employed. ***P < 0.001; ****P < 0.0001.

Therefore, we further performed GO analysis on the cluster 1 genes and found that a total of 18 apicoplast genes were enriched (Fig. 6A; *P*-value < 2.11E-11). These genes showed the highest expression in the G1 phase (5–9 hours) and the lowest expression in the S and M phases (Fig. 6B and C). Most of these apicoplast genes (9/18) are key genes of the type II fatty acid synthesis (FASII) pathway, including acetyl-CoA carboxylase (ACC1), enoyl-acyl carrier reductase (ENR), apicoplast triosephosphate translocator (APT1), acyl carrier protein (ACP), glycerol 3-phosphate acyltransferase (ATS1), and four pyruvate dehydrogenase complex subunits (PDH-E2, PDH-E1α, PDH-E1β, and PDH-E3I) (Fig. 6B and C) (42, 43). The FASII pathway is a *de novo* synthesis pathway for fatty acids (FAs) located in the apicoplast of *T. gondii*, and various components in this pathway are crucial for apicoplast formation and parasite replication (19, 42–44). Since the deletion of TgAP2XII-9 leads to the downregulation of many apicoplast genes, particularly those in the FASII pathway, a further desire was made to verify whether apicoplast division would be affected. By using IFAs conducted with anti-*T. gondii* ACP and ENR (apicoplast markers) antibodies, we found that the iKD TgAP2XII-9 parasite’s apicoplast exhibited division disorders after 24 hours of treatment with IAA (Fig. 6D).

Two different types of unnatural divisions were mainly observed as follows: (i) loss of apicoplasts. Tachyzoites in the same PV exhibited both persistence and absence of apicoplasts and (ii) apicoplast division disorder. Tachyzoites in the same PV showed different numbers of apicoplasts, including 0, 1, 2 and 3. Usually, the single copy apicoplast undergoes elongation and scission and divides equally into two daughter cells, but we observed disordered inheritance of apicoplasts in the daughter cells. For instance, one daughter cell had two or three apicoplasts, while the other had none (Fig. 6D). Quantification analysis demonstrated that 5% of vacuoles exhibited loss of apicoplasts after deletion of TgAP2XII-9 (Fig. 6E), and the frequency of vacuoles displaying two apicoplasts was significantly higher in a parasite with/without budding (Fig. 6E), with loss of apicoplast (1Nucl, 0 APC) also being noted. The same results were observed using the ENR antibody (Fig. S2A). The proportion of elongated apicoplasts was also quantified; however, no significant difference was observed (Fig. S2B). Since segregation of apicoplasts into daughter parasites occurs only after centriole duplication (8), the division of the centriole was also observed by IFA using an anti-centrin1 antibody. Besides, the results showed that the division of the centriole was not affected after TgAP2XII-9 deletion (Fig. 6F and G). Additionally, the TgAP2XII-9 did not directly bind to the promoters of the apicoplast genes, as indicated from our CUT&Tag results. Thus, these results indicate that TgAP2XII-9 plays an indirect but crucial role in the accurate division and inheritance of apicoplasts to daughter parasites. To further investigate whether abnormal disorganized apicoplast division leads to defective mitochondrial segregation, we endogenously tagged the mitochondrial localized TgF1β (TGME49_261950) in the iKD TgAP2XII-9 strain. The segregation of the mitochondria was observed by IFA using anti-Ty antibody with or without IAA treatment for 24 hours, and the results showed that the division of the mitochondria was not disturbed after depletion of TgAP2XII-9 (Fig. S2C and D).

Notably, a set of genes related to DNA duplication were downregulated upon TgAP2XII-9 deletion, with an expression pattern consistent with the that of cluster 1 genes that are peaked in expression in the G1 phase (6–9 HR) (Fig. S3). These genes were also not affected by CUT&Tag enrichment, suggesting that TgAP2XII-9 may have an indirect role in regulating parasite DNA replication, but further validation is needed.

## DISCUSSION

*T. gondii* is an obligate intracellular apicomplexan parasite that infects almost all warm-blooded animals, including humans and livestock, and causes severe secondary infections in immunocompromised populations and reproductive disorders in pregnant women. It is estimated that about one-third of the global population is infected with *T. gondii* (1, 45, 46). *T. gondii* has a complex life cycle, including sexual and asexual stages. The tachyzoites can replicate rapidly by endodyogeny, and their cell cycle is divided into three phases (G1, S, and M phases) (6, 47). The rapid and smooth transformation of the parasite cell cycle relies on precise and complicated gene regulation; however, the mechanism of parasite cell cycle regulation is not fully understood. Twenty-four ApiAP2 transcription factors in *T. gondii* are predicted to be potentially involved in cell cycle regulation, including TgAP2XII-9. Here, we demonstrate that TgAP2XII-9 has a dynamic expression pattern during the parasite cell cycle, with peak expression in the S/M phase and a minimum level in the G1 phase. This study also shows that TgAP2XII-9 is essential for the growth of tachyzoites and that TgAP2XII-9 depletion leads to a significant repression of parasite invasion, replication, and egress.

TgAP2XII-9 depletion also led to the downregulation of large amounts of cell cycle-regulated genes, including IMCs and ROPs/RONs, which are highly expressed in the S/M phase (Fig. 4B and 5A). Similarly, TgAP2X-5 deficiency resulted in the downregulation of genes highly expressed in the S/M phase (including multiple rhoptries and micronemes proteins), leading to a decrease in the virulence and invasive capacity of the parasite (7). Another study showed that knockdown of AP2X-4 resulted in the downregulation of several rhoptry proteins, especially those genes related to tissue cyst formation (ROP5, ROP17, ROP7, ROP2, ROP8, and ROP16), suggesting that AP2X-4 may affect parasitism by regulating these rhoptry proteins (16, 48). Interestingly, our results indicate that TgAP2XII-9 directly controls the expressions of RON and ROP proteins (peak expression in the S- and M- phase), including RON2, RON4, RON5, and RON8, which are important components of the moving junction (28). MJ is a key structure for host cell invasion and is formed by intimate contact between the apical tip of the tachyzoite and the host cell membrane (28). The MJ of *T. gondii* tachyzoites consists of RON2, RON4, RON5, and RON8 secreted from rhoptry necks and apical membrane antigen 1 (AMA1) secreted from micronemes (28). RON2 and RON5 are crucial for the invasion process of tachyzoites, and parasites lacking RON2 or RON5 cannot survive (29, 49). The invasive ability of RON8- and RON4-depleted parasites is significantly reduced by 70% and 60%, respectively (50, 51). In addition, AMA2 and AMA4 were found to be significantly downregulated in TgAP2XII-9-depleted parasites. AMA2 and AMA4 were found to be homologous to AMA1, but consist of a bradyzoite-specific MJ machinery and contribute to cyst burden (30). These results suggest that TgAP2XII-9 may affect the invasion of both tachyzoites and bradyzoites by regulating the transcription of MJ-related RON or AMA genes.

Apart from the RONs, we also identified seven ROPs that were directly regulated by TgAP2XII-9, namely, ROP1, ROP4, ROP15, ROP17, ROP39, ROP47, and ROP48. A subset of these proteins has been fully characterized as virulence factors interacting with host cells. ROP1 was the first rhoptry protein to be identified and is essential for parasite virulence *in vitro* and *in vivo*, as well as crucial for counteracting interferon gamma-mediated innate immune restriction (31). ROP1 also binds to host complement component 1q binding protein (C1QBP), which is a regulator of autophagy and innate immunity (31). ROP17, an active rhoptry kinase localized on the external surface of the PVM, forms complexes with the key virulent factors ROP5 and ROP18. ROP17 phosphorylates host cell IRGs (immunity-related GTPases) recruited to the PVM and leading to its disruption and parasite death (32) and inhibits the polymerization of IRGs as well as contributing to the virulence of *T. gondii* (33). ROP39 is another *T. gondii* virulence factor associated with ROP5B, which directly targets host Irgb10 and inhibits homodimer formation of GTPase by reducing IRG protein loading onto the PVM (34). ROP47 is secreted from the rhoptry bulb and relocalizes to the host cell nucleus and may play a role in manipulating host cell gene transcription (34). However, deletion of ROP47 or ROP48 in a type II strain did not show a major influence on *in vitro* growth or virulence in mice (35). Generally, these results demonstrate that TgAP2XII-9 controls the expression of parasite virulence factors, which suggests that TgAP2XII-9 contributes to parasite invasion and virulence.

Notably, the transcription levels of a large number of IMC genes with peak expression in the S/M phases and minimum expression in the G1 phase are affected by TgAP2XII-9. IMC is a unique organelle that plays many important roles in the complex life cycle of apicomplexan parasites. IMC serves as a scaffold for daughter cell assembly and is crucial for maintaining the structural stability of tachyzoites (2, 52). Depletion of TgAP2XII-9 leads to the downregulation of 52 IMC proteins, some of which are known to be localized to daughter tachyzoites (37–41, 53). Interestingly, IMC29, IMC30, IMC31, IMC44, BCC3, and IMC36 were found to be localized only in the daughter buds of parasites, whereas AC12 and AC13 are located only in the apical cap of daughter buds (37). Of these, IMC29 has been proven to be an important early daughter bud component for replication, and parasites lacking IMC29 exhibit severe growth replication defects and loss of virulence (37). IMC subcompartmental proteins (ISPs) have been used to delineate the various sub-compartments of the IMC through parasite division (39, 54). TgISP1 is present in the apical cap of both mother and daughter parasites, which is one of the first markers seen at initiation of daughter cell construction. TgISP2 and TgISP4 are localized in the central section of the IMC, and TgISP2 plays an important role in regulating cell division in *T. gondii* (39, 52, 54, 55). In addition, after depletion of TgAP2XII-9, the transcription levels of cytoskeletal proteins expressed at the later stages of the replication process were also downregulated, including IMC1, IMC3, IMC4, IMC6, and IMC10. However, IMCs (IMC7, IMC12, IMC14, IMC37, and IMC40) expressed only in the mother cells were not affected (37, 56, 57). Subsequently, the disorganization exhibited by TgAP2XII-9-depleted parasites in daughter cell division was validated using IMC1 as the marker. However, not all of the above IMCs were directly controlled by TgAP2XII-9, but many of the directly controlled IMCs (IMC3, IMC4, IMC10, IMC31, IMC34, AC12, and AC13) were restricted by daughter cell formation or endodyogeny. In summary, these results suggest that TgAP2XII-9 may affect daughter division in tachyzoites by regulating the transcription of IMC genes related to daughter buds.

In addition, one of the important roles of the IMC is to act as an anchor for the actin–myosin motor complex, which is necessary for both parasite invasion and egress (58, 59). GAP40 and GAP50 are glideosome‐associated proteins that stably anchor the motor complex to the IMC (52, 60, 61), which is significantly downregulated in TgAP2XII-9-deficient parasites and is directly controlled by TgAP2XII-9. GAP50 is firmly immobilized in the IMC and acts as a fixed anchor for the motor complex (60, 62). GAP40 has been shown to interact with components of the motor complex and is expressed concurrently with GAP50 in early daughter cells, suggesting that GAP40 may also play a role in anchoring the motor complex (52, 60, 63). Therefore, in addition to ROP and RON proteins, the downregulation of the glideosome‐associated proteins GAP40 and GAP50 may also be one of the reasons for the deficiency of parasite invasion and egress by TgAP2XII-9 knockdown.

TgAP2IX-5 has been shown to bind to the promoter of five ApiAP2s, including TgAP2XII-9, and control a large number of IMC and apical complex genes (17). We compared the RNA-seq data of TgAP2IX-5 and TgAP2XII-9 and found that part of the IMCs were downregulated in TgAP2IX-5 or TgAP2XII-9 knockdown parasites, including IMC1, IMC3, IMC6, IMC10, IMC29, IMC30, GAP40, AC2, AC4, AC7, and AC13 (17). Among these IMCs, the promoters of IMC3 and AC2 were shown to bind directly to TgAP2IX-5 and TgAP2XII-9 (17), respectively, which may indicate that TgAP2XII-9 not only acts as a downstream factor of TgAP2IX-5 but also co-opts with TgAP2IX-5 to activate the expression of IMC genes. However, this is not supported by the interactome data, which TgAP2IX-5 is not co-precipitated with TgAP2XII-9. Unlike TgAP2IX-5, the CUT&Tag data showed that TgAP2XII-9 did not bind to promoters of other ApiAP2s, even 11 AP2 downregulated and 13 upregulated. Additionally, three ApiAP2s (TgAP2XII-4, TgAP2XII-1, and TgAP2X-7) were shown to interact with TgAP2XII-9. These results may suggest indirect regulation of these AP2 factors and their downstream target genes.

Recent studies have demonstrated that histone deacetylase (HDAC3) and MORC interact with at least 12 ApiAP2s and act as a transcription repressor complex to suppress large amounts of genes (64). TgAP2XII-1 and TgAP2XI-2 have been shown to bind to the promoters of merozoite-specific genes as heterodimers and recruit MORC and HDAC3 to suppress these genes at the tachyzoite stage (64–67). The transition from the tachyzoite to merozoite stage and the division transition from endodyogeny to endopolygeny were achieved by knockdown of these factors (64–67). Notably, double knockdown of TgAP2XII-1 and TgAP2XI-2 led to a near-complete stage transition (66, 67). In contrast, TgAP2XII-2 (another MORC-interacting ApiAP2) shows highly coordinated gene targets with MORC and HDAC3 (20, 21). However, its depletion activates only merogony gene expression, but not merogony or endopolygeny (21, 67). Apart from the MORC-HDAC3-ApiAP2s gene suppression complex, knockdown of cell cycle-regulated TgAP2IX-5 results in flexibility of cell cycle patterns from endodyogeny to endopolygeny (17). Chromatin immunoprecipitation results do not support direct binding of TgAP2IX-5 to merozoite-specific gene promoters but do support a large number of IMC genes and five ApiAP2s (TgAP2IV-4, TgAP2III-2, TgAP2XII-9, TgAP2X-9, and TgAP2XII-2) (19). Therefore, assuming a cascade ApiAP2 transcription regulation model and taking into account the unsuccessful inducement of merogony due to TgAP2XII-2 depletion (67), another key factor may exist in the remaining TgAP2IX-5 targeted to ApiAP2s.

In this study, some endopolygeny was also observed after TgAP2XII-9 knockdown using TgIMC1 and TgGAP45 as staining markers. However, the abnormal divisions of daughter cells, including nonsynchronous divisions and the presence of an odd number of tachyzoites in a PV, dominated the IAA-treated population. After TgAP2XII-9 knockdown, we also found that 11% of DEGs were differently transcribed for EES genes (996 in total)(68), but these genes showed both downregulation (97/208) and upregulation (111/208). In contrast, TgMORC depletion resulted in >80% EES gene upregulation (64), and TgAP2XII-1 and TgAP2XI-2 knockdown also upregulated about half of the merozoite specific genes (65–67). Additionally, we stained TgAP2XII-9 knockdown parasites with the merogony marker TgGRA11b, but observed negative signals (data not shown). Therefore, we speculate that TgAP2IX-5 controls the parasite division pattern from endodyogeny to endopolygeny through TgAP2XII-9, but not the transition from tachyzoites to merozoites, as in the case of the MORC–HDAC3–AP2s complex dose (64–67). Also, the EES gene alteration may result by the potential interaction between TgAP2XII-1 and TgAP2XII-9, as shown by the interactome data.

Previous studies have revealed that subcellular organelles are strictly coordinated during tachyzoite replication, which invariably proceeds in the following order: centriole and Golgi → apicoplast → nucleus and ER → IMC; rhoptries and micronemes are synthesized *de novo* in each daughter cell (8). Our results indicate that deletion of TgAP2XII-9 results in significant downregulation of the transcriptional levels of many apicoplast genes, which have peak expression in the G1 phase and lowest expression in the S/M phases. The peak expression of these apicoplast genes occurred significantly earlier than those of the IMC and ROP genes regulated by TgAP2XII-9, which is consistent with the previously reported theory that the division of apicoplasts occurs earlier than that of IMC and ROP during the division cycle of tachyzoites (8). This study subsequently analyzed the functions of these TgAP2XII-9-regulated apicoplast genes and found that half of these genes are key genes involved in the process of the apicoplast FASII pathway, including ACC1, ENR, APT1, ACP, ATS1, and the four PHDs (42, 43). FASII is a *de novo* fatty acid synthesis pathway restricted in the apicoplasts of *T. gondii* and is essential throughout the tachyzoite life stage, as it provides the bulk of the fatty acids required for the synthesis of major membrane lipid classes (19, 42, 44). Dihydroxyacetone phosphate and phosphoenolpyruvate (PEP) were imported into the apicoplast through APT1 to provide fuel for the FASII pathway (19, 42, 69). The imported PEP was converted by the pyruvate dehydrogenase complex into pyruvate and ultimately into acetyl CoA. Acetyl-CoA was then carboxylated by ACC1 to form malonyl-CoA (42, 43, 70, 71). Following the ACC1 activity, acetyl-CoA and malonyl-CoA were transferred to acyl carrier proteins, which transferred the nascent fatty acid chains to different enzymes in the FASII pathway (19, 42, 43, 70). A previous study showed that specific inhibition of the FASII pathway with triclosan affects apicoplast inheritance and parasite division by preventing cytokinesis completion, resulting in incomplete daughter cell budding (72). Multiple key components in the FASII pathway have been shown to be crucial for the formation of apicoplasts and the division of daughter parasites. ACP deficiency has been proven to result in defective apicoplast biogenesis and a consequent loss of the organelle (44). In addition, disruption of TgATS1 causes defects in organelle and daughter parasite development (42). We further confirmed that the apicoplast of the IFA-based TgAP2XII-9 parasites exhibits division disorder and organelle loss. Furthermore, abnormal budding and division of the daughter parasites were observed in TgAP2XII-9-deficient parasites (Fig. 5). These results indicate that TgAP2XII-9 deficiency leads to the downregulation of multiple apicoplast genes in the FAS II pathway, which in turn affects the division/formation of apicoplasts and further causes abnormal budding and division of tachyzoites.

### Conclusion

Our findings demonstrate that TgAP2XII-9 plays a pivotal role in activating the S/M-specific cell cycle program and influencing parasite invasion and division by directly targeting the MJ and IMC genes and that TgAP2XII-9 knockdown also disturbs apicoplast inheritance in *T. gondii* tachyzoites. This insight into asexual cell cycle regulation could help provide potential therapeutic targets and enhance our understanding of *T. gondii* cell cycle dynamics.

## MATERIALS AND METHODS

### Parasites and cell culture

*T. gondii* RH Δku80 strain and derivative strains were continuously cultured *in vitro* with human foreskin fibroblasts (HFFs; ATCC, Manassas, VA, USA) or African green monkey kidney cells (Vero cells, a gift from Prof Qun Liu, China Agricultural University) using Dulbecco’s modified Eagle medium (DMEM, Macgene, Beijing, China) with 2% fetal bovine serum (FBS) (TransGen Biotech, Beijing, China) at 37°C and 5% CO_2_. Cells were cultured in DMEM supplemented with 10% FBS and incubated at 37°C in a 5% CO_2_ environment.

### Generation of transgenic *T. gondii* strains

The EuPaGDT library was employed to design the corresponding guide RNAs for the gene-specific CRISPR-Cas9 plasmid used in this experiment. The construction of the CRISPR/Cas9 plasmid was based on previously published cassettes and performed accordingly (73). Briefly, Cas9 upstream and downstream fragments containing gRNA sequences were amplified and ligated with a seamless cloning kit (Vazyme Biotech, Co., Ltd, Nanjing). To construct iKD TgAP2XII-9 parasites, the mAID sequence and a 3 × HA epitope tag were fused to the C-terminus of AP2XII-9. A plasmid (mAID-3HA-CAT-TIR1-3Flag) containing the chloramphenicol resistance gene (CmR) and the TIR1-3Flag expression cassette was constructed based on the template of our previously published work (27). For the C-terminal epitope tagging TgAP2XII-9, 59-bp PCR primers containing 39-bp fragments upstream of the TgAP2XII-9 translation stop codon and downstream of the gRNA site were designed to amplify PCR products from the mAID-3HA-CAT-TIR1-3Flag plasmid. The homologous recombination plasmid and the corresponding CRISPR-Cas9 plasmid were co-transfected into RH ΔKu80 parasites and screened in chloramphenicol-containing medium. Monoclonal parasites were identified using PCR and IFA. The degradation of AP2XII-9 was induced by IAA at a final concentration of 500 µM.

Using CRISPR-mediated endogenous tagging, we introduced a C-terminal 3 × TY epitope into TgF1β (TGME49_261950) in the iKD TgAP2XII-9 parasites. The parasites were co-transfected with the CRISPR/Cas9 plasmid and the 3TY-DHFR cassette prepared in the form of purified PCR products and selected with 3 mM pyrimethamine at 24 hours after electroporation and recovery. Transfected parasites were cloned by limiting dilution and confirmed by PCR and IFA. The primers used in this study can be found in Table S3.

### Immunofluorescence assay (IFA)

Freshly harvested parasites were inoculated onto HFF cells grown on glass coverslips in 12-well plates. After incubation, infected cells were properly fixed with 4% PFA for 1 hour, permeabilized with 0.25% Triton X-100, and then blocked with 3% bovine serum albumin (BSA) for 30 minutes. The samples were incubated with mouse anti-HA (1:500, Sigma, St. Louis, MO, USA), anti-TY (1:500), rabbit anti-TgGAP45 polyclonal antibodies (1:300), rabbit anti-TgIMC1 polyclonal antibody (1:300, an inner membrane complex marker), mouse anti-TgACP (1:300, a marker for apicoplast), mouse anti-TgENR (1:100), and rabbit anti-αCentrin 1 antibody (1:500) for 1 hour and washed three times with PBS, followed by incubation with secondary FITC- or cy3-conjugated antibodies (1:100, Proteinnovogtech, Rosemont, IL, USA) for 1 hour. Nuclear DNA was stained with Hoechst 33258 (1:100, Macgene, Beijing, China). The polyclonal antibodies for TgGAP45, TgIMC1, TgACP, TY, and TgENR were validated in the previous study (74) and were received as gifts from Professor Qun Liu in China Agricultural University; the rabbit anti-αCentrin 1 and anti-TgSAG1 antibody were gifts from Professor Shaojun Long in China Agricultural University (75).

For extracellular tachyzoite staining, parasites were grown for 24 hours in host cells, and extracellular parasites were filtered and harvested after extruded using a syringe. The purified fresh tachyzoites were then fixed with 4% PFA, and then immunofluorescence staining was performed using rabbit anti-TgIMC1 polyclonal antibodies (1:300), anti-HA (1:500, Sigma, St. Louis, MO, USA), and Hoechst 33258 (1:100, Macgene, Beijing, China). Images were obtained using a Zeiss Fluorescence Microscopy system (Zeiss, Germany).

### Intracellular replication assay

The intracellular replication effect was observed by immunofluorescence microscopy. HFF cells in 12-well plates were infected with 1 × 10^5^ fresh RH tachyzoites per well and incubated for 1 hour. The cell surface was then washed twice with PBS to remove extracellular tachyzoites, followed by incubation with IAA (500 µM) or vector (1:1000 ethanol). After 24 hours of treatment, cells were fixed with 4% PFA, and then immunofluorescence staining was performed using rabbit anti-TgGAP45 polyclonal antibodies (1:300) and Hoechst 33258 (1:100, Macgene, Beijing, China). The number of parasites per strain was determined by counting at least 100 PVs using fluorescence microscopy. Three biological replicates were assessed to determine the number of tachyzoites per PV.

### Invasion assay

The invasion assay was performed as previously described (26, 66). Briefly, parasites grown for 48 hours with or without IAA were harvested. The purified fresh tachyzoites suspended in DMEM with or without IAA were added to HFF monolayers and incubated at 37°C for 1 hour. The coverslips were then fixed with 4% PFA, and then immunofluorescence staining was performed. Extracellular parasites were stained with mouse anti-TgSAG1 polyclonal antibody (1:300) and mouse secondary FITC-conjugated antibodies (1:100, Proteintech, Rosemont, IL, USA). The cells were then permeabilized with 0.25% TritonX-100, and all parasites were stained with rabbit anti-TgGAP45 antibody and rabbit secondary Cy3-conjugated antibodies (1:100, Proteintech, Rosemont, IL, USA). The invasion efficiency was detected by two-color staining. Three biological replicates were assessed, and at least 15 random fields were counted for each replicate.

### Egress assay

Parasites were inoculated onto 12-well plates and cultured for 30 hours with or without IAA treatment. The egress was triggered with 2 µM of Ca^2+^ ionophore A23187 (Macklin, Shanghai) for 2 minutes at 37°C before fixation with 4% PFA. The IFA was performed using rabbit anti-TgGAP45 antibodies. A total of 100 vacuoles were randomly selected to count ruptured vacuoles/whole vacuoles per slide. Three independent experiments were performed.

### Plaque assay

Plaque assays were performed as described previously (76). HFFs growing in 12-well plates were infected with 200 freshly harvested tachyzoites and incubated for 7 days without disturbance. The experimental group was incubated with vector (EtOH 1:1000) or IAA (500 µM). Thereafter, infected HFFs were fixed with 4% paraformaldehyde and visualized by staining with 0.2% crystal violet solution. The plaque area was counted by pixel using Photoshop C6S software (Adobe, USA), and data were compiled from three independent experiments.

### TgAP2XII-9 recovery experiments

Parasites of the iKD TgAP2XII-9 strain were inoculated on HFF cells grown on coverslips of a 12-well plate in the presence of auxin for 16 hours. Auxin was washed off, and parasites were allowed to grow in IAA-free medium for additional 0, 3, 6, and 20 hours before being fixed with 4% PFA. Parasite nuclei were labeled with Hoechst 33258, and the parasite’s inner membrane complex (IMC) was labeled with TgIMC1. Three independent experiments were performed.

### RNA-Seq and data analysis

Transgenic parasite strains cultured in Vero cells were treated with either 500 µM IAA or vehicle for 24 hours. Total RNAs from *T. gondii* tachyzoites were then extracted using the M5 Total RNA Extraction Reagent (Mei5 Biotechnology Co., Ltd, Beijing) according to the manufacturer’s protocol. Each treatment consisted of three biological replicates. The purity, concentration, and integrity of RNAs were tested using the NanoPhotometer (IMPLEN, CA, USA), the Qubit RNA Assay Kit in Qubit 2.0 Fluorometer (Life Technologies, CA, USA), and the RNA Nano 6000 Assay Kit of the Bioanalyzer 2100 system (Agilent Technologies, CA, USA), respectively. Only qualified samples were used for library preparation. Illumina sequencing libraries were generated using the NEBNext Ultra RNA Library Prep Kit for Illumina (NEB, USA) according to the manufacturer’s recommendations. Sequencing was performed using the Illumina NovaSeq 6000 platform from Shanghai Personal Biotechnology Co., Ltd. to generate 150-bp paired-end reads. The original sequencing data can be found in the Sequence Read Archive database under the accession number PRJNA1070975.

RNA-seq clean reads were uploaded to the BMKCloud (www.biocloud.net) platform for analysis based on the reference genome of Toxoplasma Type II ME49 strain (ToxoDB-57). Briefly, paired-end clean reads were aligned to the reference genome using Hisat2 (77). Read counts were calculated for each gene using the sorted bam files from StringTie (78). Differentially expressed genes (DEGs) between treated and untreated parasites were calculated by edgeR (79). Gene ontology enrichment analysis was performed in ToxoDB. Gene expression with a fold change >2 or < −2 and FDR < 0.05 was considered as significantly differentially expressed. TPM (transcripts per kilobase million) values were calculated for each gene and used for generating clustered heatmaps. Gene clustering was performed using Mfuzz from the R package. Gene Ontology enrichment analysis was conducted utilizing topGO (80) and GO annotations accessible on ToxoDB.org (version 64), with significant GO terms identified based on a Benjamini < 0.1.

### CUT&Tag and data analysis

Transgenic strains were inoculated in 25-cm^2^ cell culture flasks and washed after 4 hours to remove noninvasive parasites. Transgenic strains (1 × 10^7^) were harvested after 20 hours of incubation. Library construction was performed using the NovoNGS CUT&Tag 4.0 High-Sensitivity Kit for Illumina (Novoprotein, Suzhou, China) according to the manufacturer’s instructions. Briefly, fresh tachyzoites were bound to activated concanavalin A beads (10 µL/sample) and incubated for 10 minutes at room temperature. The mixture was resuspended and incubated with the primary antibody (1:50, mouse anti-HA) at 4°C overnight. After several washes, the parasites were incubated with the secondary antibody (1:100, goat anti-mouse IgG) for 1  hour at room temperature. The parasites were then resuspended with pAG-transposome buffer and incubated for 1 hour at room temperature on a rotator. Tagmentation was stopped by MgCl_2_ treatment, and DNA extraction was performed using DNA extract beads (Novoprotein, Suzhou, China). Illumina sequencing libraries were generated by PCR amplification using specific adapters according to the manufacturer’s recommendations (NovoNGS CUT&Tag 4.0 High-Sensitivity Kit for Illumina B box, Novoprotein, Suzhou, China). CUT&Tag libraries were sequenced using the Illumina NovaSeq 6000 platform (Beijing Novogene Technology Co., Ltd).

The paired-end reads were filtered and then aligned to the *T. gondii* ME49 reference genome using Bowtie2 (81) (v.2.1.0). The resulting sam files were transformed into bam files. PCR duplicates were removed from the sorted bam files using Picard tools (https://broadinstitute.github.io/picard/). The filtered reads were then employed to identify CUT&Tag peaks using MACS2 (82). The overlapped peaks in the two biological replicates were identified by the Irreproducibility Discovery Rate (IDR) (83). Final peaks were annotated against the latest *T. gondii* reference genome in ToxoDB. The sorted and filtered bam files of CUT&Tag peaks and RNA-seq reads were normalized to RPKM with a resolution of 10 bp (bin size) and transformed into bigwig files for direct visualization in IGV (Integrative Genomics Viewer) (84). Raw sequencing data and processed data are available in the NCBI GEO database under the accession number GSE266204.

### Immunoprecipitation and MS-based proteomic analyses

Immunoprecipitations were conducted as previously reported (66, 85). Briefly, iKD TgAP2XII-9 parasites cultured in the medium for 48 hours were collected, syringe-filtered, and pelleted by centrifugation at 1000 × *g* for 10 minutes at 4°C. The parasite pellets were washed with cold PBS before lysation with IP lysis buffer (huaxingbio, Beijing, China) containing 1 × protease inhibitor cocktail on ice for 45 minutes. The parasite lysates were centrifuged at 12,000 *g* for 10 minutes at 4°C, and the supernatants were incubated with mouse monoclonal anti-HA-tagged protein A/G magnetic beads (MedChemExpress, Shanghai, China) on a rotator at 4°C overnight. After washing, the bound proteins were eluted from the magnetic beads using the elution buffer provided by the kit. The eluted proteins were prepared with SDS-PAGE Sample Loading Buffer and heated at 95°C for 5 minutes. Subsequently, the samples were digested by trypsin at 37°C for 4 hours, and the resulted peptides were detected by the Vanquish Neo UHPLC -Astral LC/MS DIA method by using the Vanquish Neo upgraded UHPLC system (Thermo) and orbitrap astral mass spectrometer (Thermo) at Beijing Novogene Technology Co., Ltd. The resulted data were searched against *T. gondii* database (Type II ME49 strain, ToxoDB-57) using the DIA-NN library search software. Only credible spectral peptides and proteins were retained, and FDR validation was performed to remove peptides and proteins with an FDR greater than 1%. A protein with a |fold change (FC)| >1.2 and *P*-value < 0.05 was defined as a differentially expressed protein.

### Statistical analysis

Violin charts, line drawings, scatter plots, and histograms were generated using GraphPad Prism 9 (San Diego, CA, USA). Heatmaps were drawn using the OmicStudio tool (86) on https://www.omicstudio.cn/tool. Time series analysis was performed online in BioLadder (bioladder.cn). All experiments were performed in independent biological replicates as described for each experiment in the manuscript. Statistical significance in plaque assay, invasion, proliferation, and parasite growth inhibition assay was evaluated by two-tailed unpaired *t*-tests or two-way ANOVA using GraphPad Prism. Statistical data are expressed as mean value ±standard error.

## Data Availability

RNA-Seq data generated in this study have been deposited to the NCBI Sequence Archive (SRA) under the accession number PRJNA1070975. Raw sequencing data and processed data for CUT&Tag experiments are available in the NCBI GEO database under the accession number GSE266204.

## SUPPLEMENTAL MATERIAL

Fig. S1 mbio.01336-24-s0001.tif Venn diagram for the differently expressed genes and enteroepithelial-stage-specific genes.

Fig. S2 mbio.01336-24-s0002.tif TgAP2XII-9 deficiency affects the division of apicoplast.

Fig. S3 mbio.01336-24-s0003.tif TgAP2XII-9 regulates DNA replication in tachyzoites.

Table S1 mbio.01336-24-s0004.xlsx Integrated CUT&Tag hits and transcriptomic data for the iKD TgAP2XII-9 strain.

Table S2 mbio.01336-24-s0005.xls MS-based proteomic analyses of the potential interactome of TgAP2XII-9.

Table S3 mbio.01336-24-s0006.xlsx Primers used in this study.

ASM does not own the copyrights to Supplemental Material that may be linked to, or accessed through, an article. The authors have granted ASM a non-exclusive, world-wide license to publish the Supplemental Material files. Please contact the corresponding author directly for reuse.
