# Supplementary figures and images for: Cell cycle-regulated transcription factor AP2XII-9 is a key activator for asexual division and apicoplast inheritance in Toxoplasma gondii tachyzoite
Source: mBio. 2024 Aug 29;15(10):e01336-24. doi: 10.1128/mbio.01336-24 (PMC11481911; doi:10.1128/mbio.01336-24)

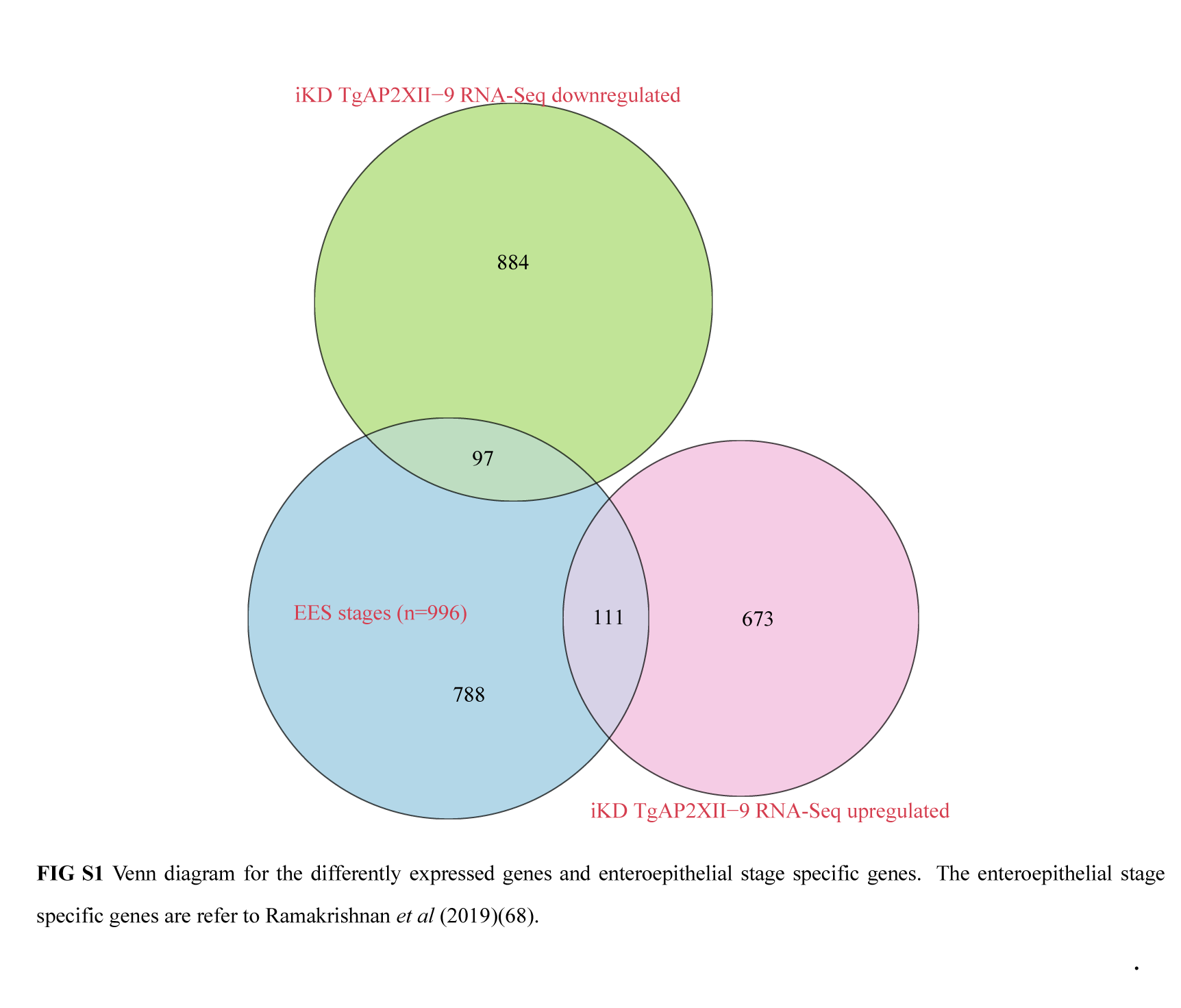

Supplement: Fig. S1 — Venn diagram for the differently expressed genes and enteroepithelial-stage-specific genes. [file mbio.01336-24-s0001.tif]

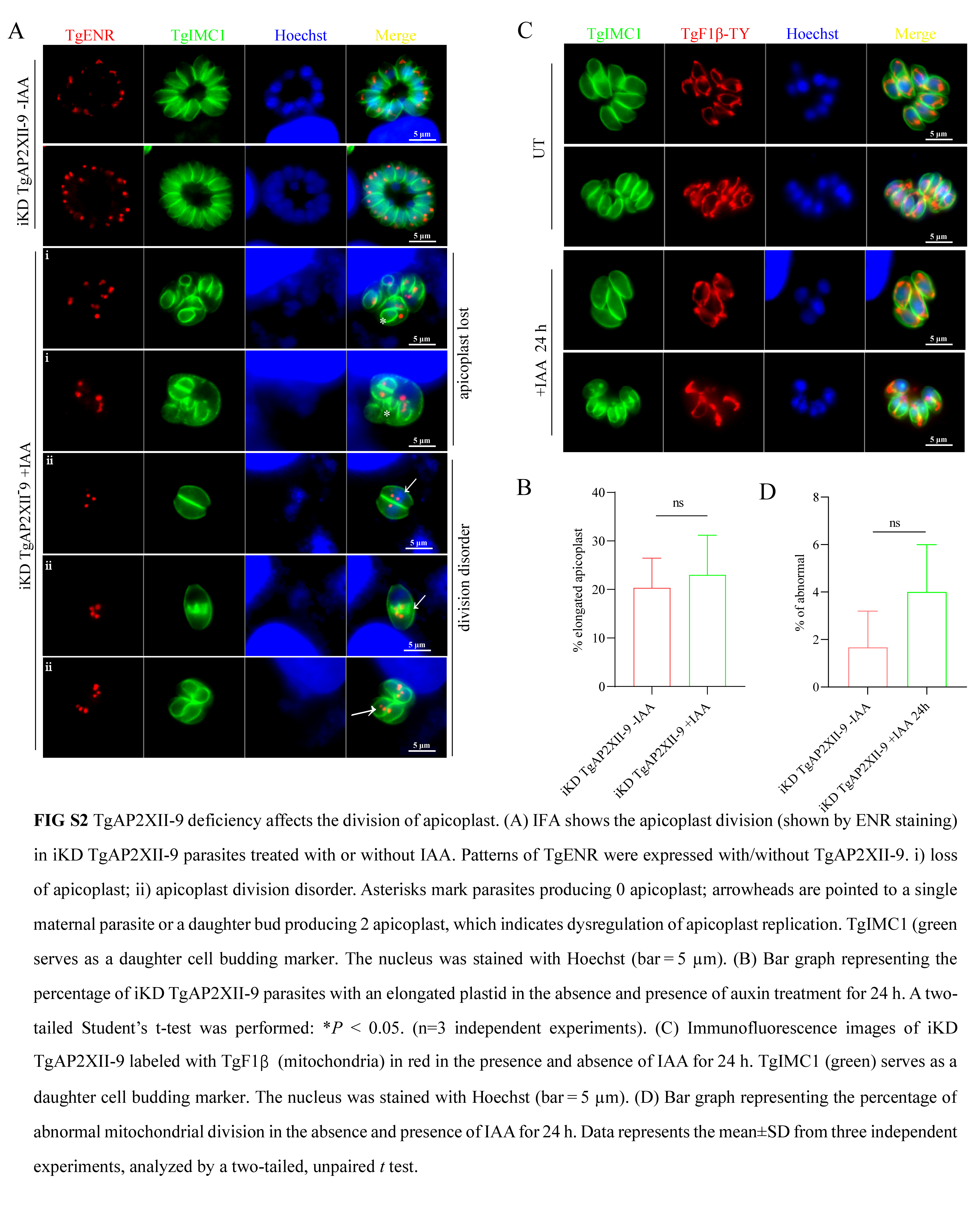

Supplement: Fig. S2 — TgAP2XII-9 deficiency affects the division of apicoplast. [file mbio.01336-24-s0002.tif]

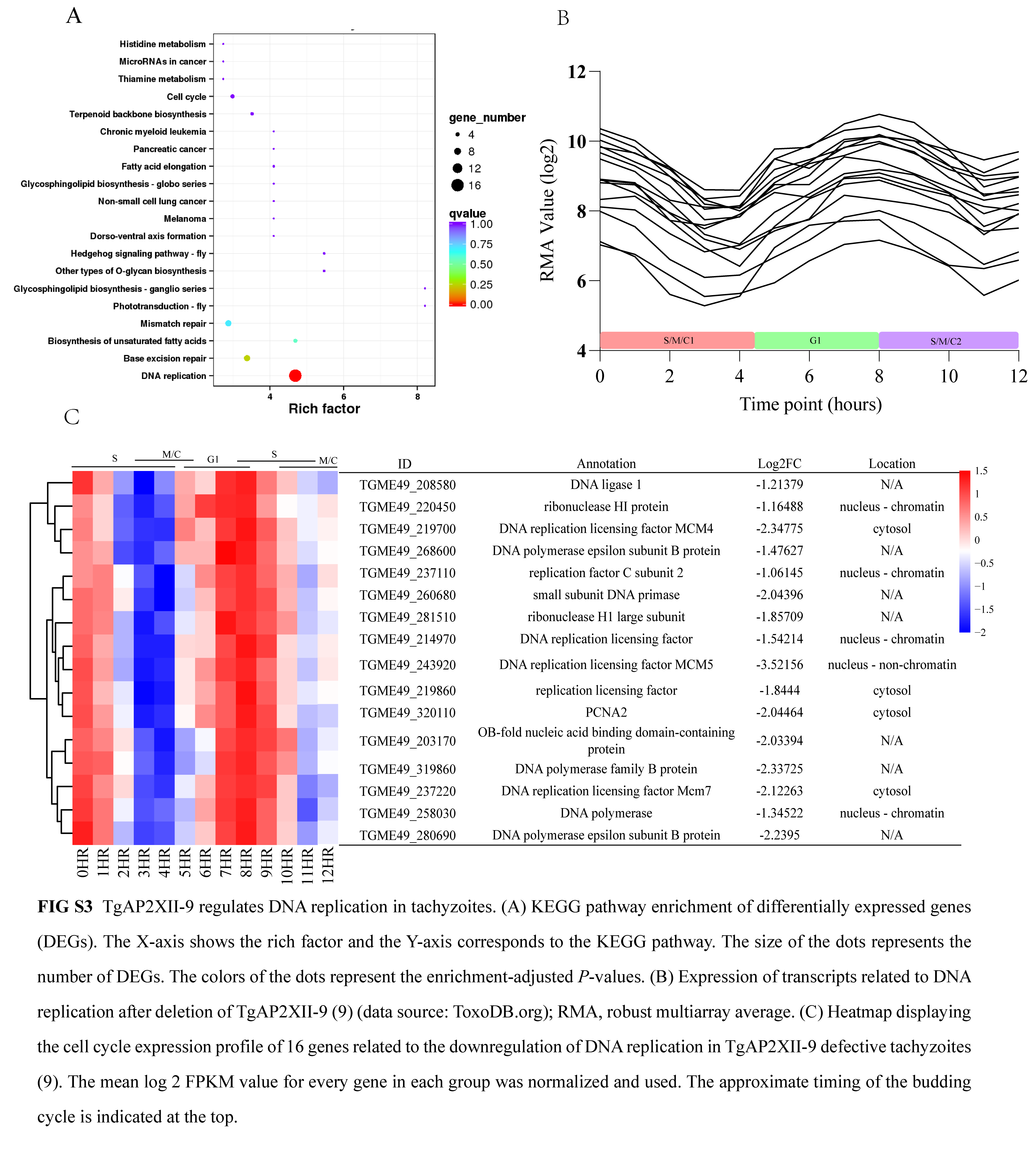

Supplement: Fig. S3 — TgAP2XII-9 regulates DNA replication in tachyzoites. [file mbio.01336-24-s0003.tif]
